# Integrated Droplet-Based Digital Loop-Mediated Isothermal Amplification Microfluidic Chip with Droplet Generation, Incubation, and Continuous Fluorescence Detection

**DOI:** 10.3390/bios14070334

**Published:** 2024-07-08

**Authors:** Yen-Heng Lin, Yuan-Ting Hung, Wei Chang, Chiuan-Chian Chiou

**Affiliations:** 1 Department of Biomedical Engineering, Chang Gung University, Taoyuan 333, Taiwan; 2 Department of Laboratory Medicine, Chang Gung Memorial Hospital, Linkou, Taoyuan 333, Taiwan; 3 Department of Electronic Engineering, Chang Gung University, Taoyuan 333, Taiwan; 4 Master and PhD Program in Biotechnology Industry, College of Medicine, Chang Gung University, Taoyuan 333, Taiwan; deamit114@gmail.com; 5 Department of Medical Biotechnology and Laboratory Science, College of Medicine, Chang Gung University, Taoyuan 333, Taiwan; 6 Graduate Institute of Biomedical Sciences, College of Medicine, Chang Gung University, Taoyuan 333, Taiwan

**Keywords:** droplet, digital LAMP, microfluidics, staphylococcus aureus

## Abstract

This study integrated sample partition, incubation, and continuous fluorescence detection on a single microfluidic chip for droplet-based digital Loop-Mediated Isothermal Amplification (LAMP) of nucleic acids. This integration eliminated the need to transfer reactions between different platforms, avoiding sample contamination and loss. Prior to the reaction, filling the channels with an oil phase and adding a glass cover slip on top of the chip overcame the problem of bubble generation in the channels during the LAMP reaction due to heating. Additionally, using two fluorescence intensity thresholds enabled simultaneous detection and counting of positive and negative droplets within a single fluorescence detection channel. The chip can partition approximately 6000 droplets from a 5 µL sample within 10 min, with a droplet diameter of around 110 µm and a coefficient of variation (CV) value of 0.82%. Staphylococcus aureus was quantified via the proposed platform. The results demonstrated a highly accurate correlation coefficient (R = 0.9998), and the detection limit reached a concentration of 1.7 × 10^2^ copies/µL. The entire process of the droplet digital LAMP reaction, from droplet generation to incubation to quantitative results, took a maximum of 70 min.

## 1. Introduction

Nucleic acid amplification testing (NAAT) is a molecular biology technique used to detect and identify pathogenic microorganisms, viruses, and genetic mutations by duplicating nucleic acid sequences in a sample. Common NAAT methods include Polymerase Chain Reaction (PCR), Loop-Mediated Isothermal Amplification (LAMP), and Recombinase Polymerase Amplification (RPA). PCR is the most widely used method [1] but requires precise temperature control equipment. LAMP, established in 2000 by Notomi et al. [2], uses a strand-displacing *Bst* DNA polymerase and at least two sets of primers. Due to the design of the primers and the unique properties of the *Bst* DNA polymerase, the amplification process can be conducted at a constant temperature (usually 60–70 °C) without the thermal cycling required for PCR, which produces about 1000 times more product, making it a promising nucleic acid amplification technique [3,4,5]. Like PCR, LAMP can also be coupled with fluorescence to form quantitative LAMP (qLAMP), which allows for semi-quantitative analysis through time threshold (Tt) values. However, many studies have noted that LAMP shows greater variability in Tt values at lower concentrations [6,7,8] of the sample, as it does not initiate each DNA amplification synchronously with temperature, allowing all biochemical reactions to occur simultaneously. These reactions can affect Tt values due to any kinetic changes in the reaction processes. Digital LAMP (dLAMP) avoids these issues, as it belongs to endpoint detection and uses positive events to calculate the original sample concentration. dLAMP involves partitioning the sample into multiple independent reaction chambers, with each nucleic acid sample randomly distributed into the chambers before the LAMP reaction is carried out. Additionally, digital LAMP can prevent low-concentration analytes from being overshadowed or interfered with by dominant DNA during the reaction, enabling the detection of low-copy number samples and increasing sensitivity.

Microfluidic dLAMP chip development generally falls into two categories: chamber-based dLAMP and droplet-based dLAMP. Chamber-based microfluidic dLAMP typically involves using photolithography or chemical etching to create thousands or tens of thousands of uniformly sized microchambers on a chip. Sample input methods include using positive or negative pressure to fill all chambers and channels on the chip, then introducing an oil phase into the chip’s channels to replace the sample and independently isolate each chamber [9,10,11], or using pneumatic microvalves (Quake valves) to segregate each chamber [12]. Another approach involves using slip chips, where the relative movement of the top and bottom plates of the chip uniformly fills independent microchambers [13,14]. Overall, the sampling method for chamber-based microfluidic dLAMP is straightforward, but the manufacturing threshold for microchambers is high, requiring a clean room and multilayer photolithography processes. On the other hand, the manufacturing threshold for droplet-based microfluidic dLAMP chips is low [15,16,17]. In the chip, T-junctions or cross-sections [18] with microchannels are created using oil as the continuous phase and the sample as the dispersed phase. By setting the flow rate ratio of oil to water, droplets of different sizes can be formed. This type of microfluidic chip can be produced using a single-layer photomask in photolithography, which is simpler than the multilayer masks required for different channel and chamber heights in chamber-based microfluidic dLAMP or the multilayer PDMS needed to form pneumatic microvalves. In addition, during the LAMP reaction, the partitioning number and volume can be adjusted by changing the flow rate of oil and water, thus enhancing the detection sensitivity and dynamic range. This study used droplets to partition samples.

After completing the sample partition in a droplet-based microfluidic dLAMP, the next steps are incubation and detection. In terms of droplet detection technology, most studies use two techniques: imaging analysis technology [19,20,21] and single droplet detection technology [22,23,24]. Imaging analysis technology involves exciting all droplets with a light source, capturing images with a CCD camera, and analyzing the droplets. The advantage of imaging analysis is its fast analysis speed; however, it requires avoiding droplet overlap and stitching images together due to the lens’ limited field of view. The principle of continuous single droplet detection is similar to flow cytometry with its laser-induced fluorescence detection technology, where a light source illuminates each droplet, and a light sensor receives the fluorescence signal from each droplet. This method has the advantages of high sensitivity and the ability to obtain comprehensive droplet information. However, continuous individual droplet detection technology cannot be easily integrated into droplet-based digital LAMP or PCR microfluidic chips. In addition, most droplet-based assays with single droplet detection technologies require three separate machines to complete sample partition, incubation, and detection [25,26,27], which may lead to sample contamination or loss during transfer. In this study, we used continuous single droplet detection technology to achieve high sensitivity and integrated these three steps onto a single chip using microfluidic technology. This integration was expected to offer a more efficient and flexible platform for conducting dLAMP, enhancing its potential in various analytical and diagnostic applications.

## 2. Materials and Methods

### 2.1. Chip Design and Fabrication

**Figure 1:**
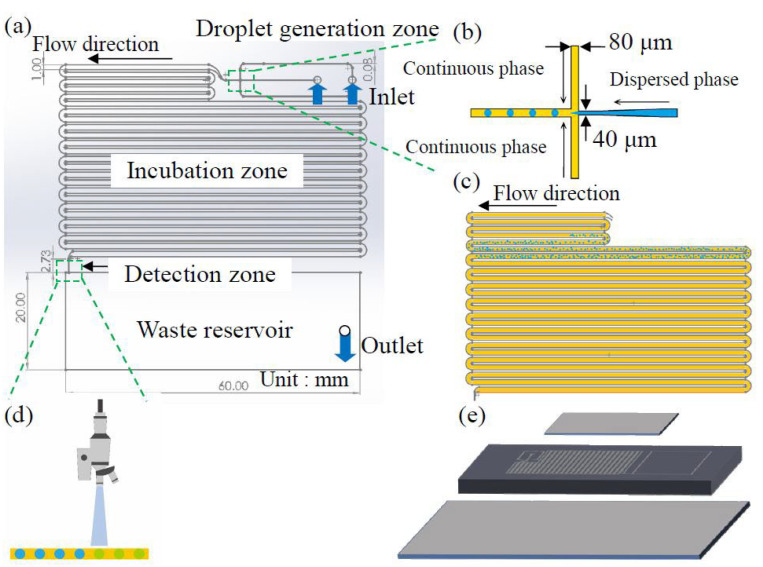
(**a**) The droplet-based digital LAMP microfluidic chip is designed to integrate droplet generation, droplet incubation, and droplet detection into a single chip. (**b**) The design features a cross-channel for micro-droplet generation, with the continuous and dispersed phases having channel widths of 80 μm and 40 μm, respectively. (**c**) The chip includes a droplet incubation zone with an incubation channel approximately 135 cm in length. (**d**) For droplet detection, a detection channel with a width of 100 μm is incorporated into the chip, allowing droplets to pass through the fluorescent detection zone individually. (**e**) An exploded view of the chip reveals its composition, which includes a thin cover glass, a PDMS microchannel layer, and a bottom glass substrate.

The microfluidic chip design shown in Figure 1a consists of four functional blocks: droplet generation zone, droplet incubation zone, droplet detection zone, and waste reservoir. The channel height is 80 µm. The droplet generation area features a cross-channel design with an oil-phase channel width of 80 µm and a water-phase channel width of 40 µm, as shown in Figure 1b. The tapered design of the dispersed phase channel produces smaller droplets while ensuring the durability of the master mold. The droplet incubation area has a channel width of 1 mm and length of 135 cm to fully retain the droplets for incubation, accommodating up to approximately 110 µL of fluid, as depicted in Figure 1c. After incubation, droplets flow into the fluorescence detection area (Figure 1d), which is 2.73 mm long and 100 µm wide, allowing only one droplet to pass at a time to ensure single droplet detection. The waste reservoir is 60 mm long and 20 mm wide, designed with a height of 300 µm to contain up to 360 µL of waste fluid, as shown in Figure 1d, to prevent sample contamination by retaining all the reacted reagents within the chip. The chip is constructed with a glass-PDMS-glass sandwich structure, as depicted in Figure 1e. It uses 0.2 mm thick glass as the substrate to facilitate heat transfer during incubation, a 0.5 cm thick PDMS layer containing the microchannel structures, and a 0.2 mm thick glass cover on top to prevent gas from entering the channels through the PDMS. As illustrated in Supplementary Figure S1, the chip fabrication process begins with the creation of the master mold through standard photolithography on a 4-inch silicon wafer using a negative photoresist (SU-8 2050, Kayaku Advanced Materials, Westborough, MA, USA) spun at 500 rpm for 10 s and 2100 rpm for 30 s. After soft baking, exposure, post-exposure baking, and development, the photomask design is transferred to the wafer, followed by a hard bake at 120 °C for 15 min. The waste reservoir, which requires less height precision than the channels, is fabricated using a dry film photoresist (Ordyl SY 355, Resistechno, Milan, Italy) rather than a second exposure, simplifying the process. The dry film is cut to the size of the waste reservoir, and four layers are applied to achieve a height of approximately 300 µm. It is then heated on a hot plate at 150 °C for 30 min to complete the master mold.

Microchannels are manufactured from polydimethylsiloxane (PDMS, SYLGARD 184 Silicone Elastomer Kit, Dow Corning, Midland, MI, USA). To minimize PDMS porosity and reduce gas permeability, a 10:2 ratio of part A to part B is used, followed by curing at 80 °C for 30 min. The cured PDMS is then peeled from the master mold, and holes are punched at the inlet and outlet. The PDMS is bonded to two glass slides with dimensions of 80 × 80 × 0.2 mm using an oxygen plasma machine (PDC-001, Harrick Plasma, Ithaca, NY, USA) set to 30 W power and 0.05 Torr oxygen pressure. Finally, the chip undergoes surface modification to facilitate the formation of water-in-oil droplets. A hydrophobic agent (Aquapel, PPG Industries, Pittsburgh, PA, USA) is introduced into the channels. Due to the wide 1 mm width of the droplet incubation area, the hydrophobic agent does not completely fill all the channels and may leave small air bubbles. These bubbles are removed by gently pressing the chip with tweezers, followed by flushing with fluorinated oil (HFE-7500, 3M, Saint Paul, MN, USA) to expel the hydrophobic agent. A pressure-based flow pump is used to introduce the hydrophobic agent and fluorinated oil at 600 mbar into the microchannel. Nitrogen gas is then used to clear fluorinated oil from the channels. The chip is left at room temperature to allow the oil to evaporate. Detailed procedures for Aquapel treatment can be found in reference [28]. Supplementary Figure S1 depicts the detailed fabrication process of the chip. The chip size is 80 × 80 mm; the assembled chip is shown in Supplementary Figure S2.

### 2.2. Experimental Setup and Reagent Preparation

**Figure 2:**
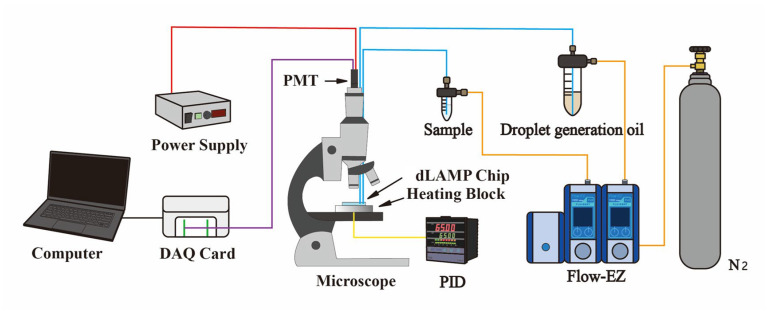
Experimental setup for the droplet-based digital LAMP microfluidic chip: The fluid flow was controlled by a pressure-based pump. A home-built temperature-controlled hot plate was integrated into the sample stage of the fluorescent microscope. The fluorescence of the droplet was detected using a microscope objective.

The experimental setup, as shown in Figure 2, consisted of three main parts: fluid control, temperature control, and fluorescence signal detection. A pressure-based flow control system (Flow EZ, Fluigent, Paris, France) was used to regulate the flow of the oil and water phases, and was connected to the microfluidic chip via Teflon tubing (1/16” O.D., 0.020” I.D., IDEX, Northbrook, IL, USA). Heat was provided by an aluminum block heater placed under the chip, equipped with a polyimide film heater (KP100100R125, MIYO Technology Co., New Taipei City, Taiwan) at the bottom of the block, and a PT100 temperature sensor embedded within it. Temperature was precisely controlled using a PID controller (F4C, Vertex Technology, New Taipei City, Taiwan), with the block heater integrated above the microscope stage. A 1 mm thick PEEK substrate was placed between the block heater and the microscope stage for thermal insulation. Fluorescence signals were detected using a photomultiplier tube (PMT, H9306-04, Hamamatsu, Japan) positioned at the C-mount interface of the microscope. The filter set in the detection system had an excitation wavelength range of 467–498 nm, an emission wavelength range of 513–548 nm, and a dichroic wavelength of 506 nm. Signals were captured through a 20X objective lens and processed in real-time using a data acquisition module (DAQ, USB-6281, National Instruments, Austin, TX, USA) and LabVIEW 2020 software (National Instruments, Austin, TX, USA). A peak detection algorithm was employed to identify and count the number of droplets using thresholds for positive and negative signals.

The target DNA amplified was from *Staphylococcus aureus*. The sample volume setup was 20 µL, with 5 µL injected into the chip for each reaction. The reagent composition included 4 µL of a home recipe LAMP buffer and 2 µL of primers (Integrated DNA Technologies, Coralville, IA, USA). The forward outer primer sequence was CCAACAGTATATAGTGCAACTTC, and the backward outer primer sequence was TTGCATTTTCTACCATTTTTTTCG at a final concentration of 0.2 µM. The forward and backward inner primer sequences were AATGTCATTGGTTGACCTTTGTACAATTACATAAAGAACCTGCGAC and GACTATTATTGGTTGATACACCTGACACTTGCTTCAGGACCATATT, respectively, at a final concentration of 1.6 µM. The forward and backward loop primer sequences were AACCGTATCACCATCAATCGC and CAAAGCATCCTAAAAAAGGTGTAGAGA, respectively, at a final concentration of 0.8 µM. The master mix of LAMP included 1 µL of *Bst* 2.0 WarmStart DNA Polymerase (New England Biolabs, Ipswich, MA, USA), 3 µL of 10 mM dNTPs, and 1 µL of 20X EvaGreen dye (Biotium, Fremont, CA, USA). EvaGreen dye is commonly used in quantitative LAMP applications. It is a nucleic acid dye that is essentially non-fluorescent on its own but becomes highly fluorescent upon binding to double-stranded DNA. When the target DNA fragment is duplicated, the fluorescent signal increases accordingly. *S. aureus* was cultivated, and its genomic DNA was purified (Presto™ Mini gDNA Bacteria Kit, Geneaid Biotech Ltd., New Taipei City, Taiwan) and quantified (EzDrop-1000, Blue-Ray Biotech, New Taipei City, Taiwan). The copy number was then calculated and diluted to the appropriate concentration. Mineral oil (MR1284-065, Tedia, Fairfield, OH, USA) was used to prevent evaporation of the reagents, and fluorinated oil (Droplet Generation Oil for EvaGreen, Bio-Rad, Hercules, CA, USA) was used for droplet generation.

### 2.3. Experimental Procedure

Firstly, 200 µL of mineral oil was injected into the chip to prevent the evaporation of fluorinated oil and the sample during the LAMP reaction. Fluorinated oil was then introduced through the oil-phase inlet to fill the incubation and detection channels, allowing the mineral oil to remain in the reservoir. The chip was left to sit for 30 min to allow the PDMS to absorb the fluorinated oil. Afterward, 5 µL of LAMP reagent was connected to the chip’s aqueous phase inlet. Both the aqueous and oil phases were introduced into the chip through a pressure-based flow control system. As the fluids passed through the cross-channel region of the chip, the dispersed phase formed uniformly sized microdroplets due to the shearing forces generated by the continuous phase. Considering that the aqueous phase reagent is encapsulated in oil, the applied air pressure was stopped to keep the droplets stationary in the incubation zone of the channel. The LAMP reaction proceeded at 65 °C for 40 min. Upon completion, the chip was immediately cooled to room temperature to terminate the LAMP reaction. Following this, the oil-phase pressure was increased to 100 mbar to continue pushing the fluorinated oil, using the oil as a carrier to transport the droplets to the detection channel. The total number of droplets and those containing fluorescence were counted as they passed through the detection channel. After all droplets were detected, the Teflon tubing and chip were placed in a Ziploc bag for disposal. The equipment and environment were cleaned with bleach.

### 2.4. Data Analysis

Quantitative DNA analysis in digital LAMP relies on a Poisson distribution to compute DNA quality based on the number of positive droplets out of the total droplets. In this process, reagent samples are divided into thousands or tens of thousands of droplets. Ideally, each droplet would randomly contain zero, one, or more copies of the target DNA. The Poisson distribution, which describes the frequency of independent events within a defined space or interval, aptly models DNA distribution across these droplets. To calculate DNA quantities, the number of positive and total droplets is input into the Poisson distribution formula.

(1) $$c=\frac{-\ln(1-\frac{b}{n})}{v}$$

Here, *c* represents the DNA concentration in units of copies/µL, *b* represents the number of positive droplets, *n* is the total number of droplets, and *v* is the volume of each droplet in µL. The volume of the droplet was estimated based on its cylindrical shape with a diameter of 110 µm and a height of 80 µm.

## 3. Results and Discussion

### 3.1. Droplet Preparation

**Figure 3:**
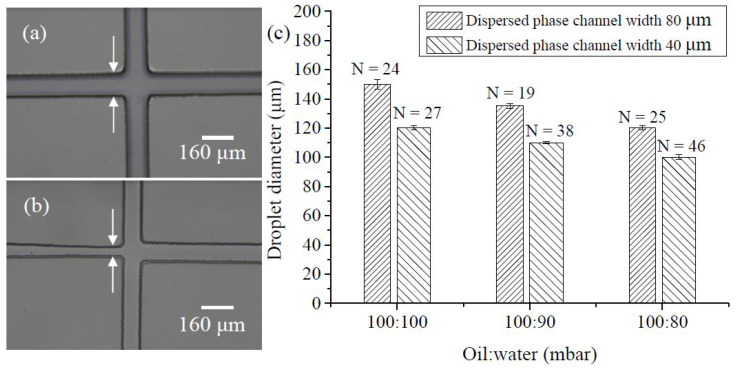
(**a**) A photograph of the cross-shaped microchannel with an 80 μm width. (**b**) The channel width of the dispersed phase is reduced to 40 μm. (**c**) The relationship between droplet size and the applied pressure ratio of oil to water generated in two different dimensions of the dispersed phase flow microchannel. The N value represents the number of droplets counted to calculate the average droplet size.

Due to the detection channel’s width of 100 µm, the target size of the droplets produced must be around 100 µm in diameter to allow droplets to pass through the detection point one by one when detecting fluorescence signals. The size of the droplets is related to the flow rates of the continuous and dispersed phases, as well as the cross-sectional area of the cross-shaped channel. Initially, the cross-shaped channel width in the continuous and dispersed phases was designed to be 80 µm. Under three different pressure ratios from the continuous to dispersed phases—100:100 mbar, 100:90 mbar, and 100:80 mbar—droplets with average diameters of 150.03 µm (C.V. = 2.10%), 135.15 µm (C.V. = 1.16%), and 120.33 µm (C.V. = 1.25%) were produced. When the aqueous phase pressure was further reduced or the oil-phase pressure increased, the oil phase flowed into the aqueous channel from the outlet of the cross-shaped channel, preventing the production of smaller droplets. Therefore, the orifice of the aqueous channel was reduced to 40 µm to increase the flow rate. Figure 3a,b show photographs of the chip with an aqueous channel with orifice sizes of 80 µm and 40 µm, respectively. The 40 µm orifice channel employed a convergent design to enhance the strength of the SU-8 structure in the mold, compared to a mold with a consistent width of 40 µm throughout the entire channel structure, increasing the number of times the mold can be reused.

Figure 3c shows the droplet sizes produced under three oil–water pressure ratios using both 80 µm and 40 µm aqueous outlet widths. After photographing and analyzing these sizes with ImageJ software (version 1.54h), an aqueous channel width of 40 µm and continuous to dispersed phase pressure ratios of 100:100 mbar, 100:90 mbar, and 100:80 mbar were found to produce droplets with average diameters of 120.32 µm, 110.07 µm, and 100.13 µm and coefficients of variation of 1.20%, 0.82%, and 1.71%, respectively. The results showed that reducing the aqueous channel width increased the flow velocity and produced smaller droplets. Regardless of the pressure ratio and channel width conditions, the droplet sizes produced by this chip were quite uniform. Since the produced droplets must be heated to 65 °C and left to sit in the incubation channel for 40 min, we found that droplets with a diameter of 110 µm were more likely to stay stable in the channel due to greater friction within the channel walls than those with a diameter of 100 µm. Since the diameter of the 110 µm droplets was larger than the height of the 80 µm microchannel, the droplets deformed into a cylindrical shape rather than a spherical shape. The droplets experienced slight friction with the top and bottom surfaces of the microchannel as they passed through the incubation zone. This friction helped the droplets remain stable within the channel. Therefore, subsequent digital LAMP experiments were conducted on droplets with a diameter of 110 µm using air pressure conditions that produced this droplet size. This method allowed about 6000 droplets to be independently partitioned from 5 µL of the sample within 10 min.

### 3.2. Droplet Stability during Incubation

**Figure 4:**
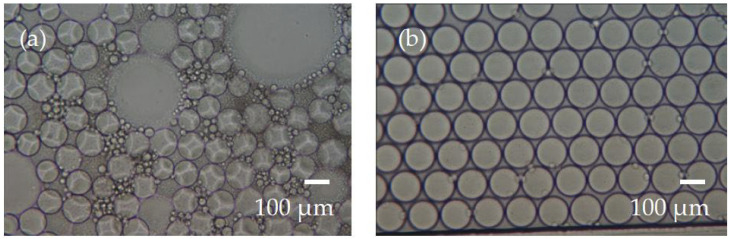
Evaluation of droplet stability during LAMP thermal incubation. (**a**) The droplets were unstable if air bubbles appeared in the microchannel. (**b**) By avoiding air bubbles in the microchannel, droplets remained intact throughout the thermal incubation period.

The LAMP reaction conditions were 65 °C for 40 min, which maintained the stability of the droplets during the incubation process without bursting or merging—one of the most crucial steps in the entire digital LAMP reaction. Initially, droplets of approximately 110 µm in diameter were produced under specified air pressure conditions for the LAMP reaction. After incubation at 65 °C for 40 min, we observed that more than half of the droplets had burst or merged with others upon microscopic examination, as shown in Figure 4a. This situation renders the digital LAMP reaction unfeasible. The experiment identified two reasons for droplet rupture. The first reason was that after the reagent was encapsulated into droplets with fluorinated oil, the end of the fluorinated oil plug did not contact the air. Otherwise, during the LAMP reaction, the end of the fluorinated oil plug that contacts the air evaporates quickly as the temperature rises. As the oil evaporates, the droplets burst. The second reason is that the PDMS itself has many microscopic pores that allow gas to permeate. During the LAMP reaction, small air bubbles gradually formed in the channels as the reagent temperature increased. These small bubbles combined into a large air gap, which promoted the evaporation of fluorinated oil and moved within the channel, leading to droplet rupture or incomplete heating.

To address these issues, the channel was filled with 200 µL of mineral oil before generating droplets. Then, droplets encapsulated in fluorinated oil were produced. We ensured that the end of the fluorinated oil plug was tightly connected to the mineral oil to prevent air contact. This method effectively improved the problem of fluorinated oil evaporation at the end of the channel during the LAMP reaction. To prevent air permeation through PDMS pores, a 0.2 mm thick glass cover was added to the top of the PDMS chip. Since glass is impermeable, it completely blocks air exchange above the microchannels through PDMS. Using this improved method for digital LAMP condition testing, droplets of approximately 110 µm in diameter were incubated at 65 °C for 40 min. While observing droplets in 10 different areas under the microscope within the fluorinated oil, we found that they had almost no bursting or merging, as shown in Figure 4b. This method allowed the droplets to remain stable in the channels during the digital LAMP process. In addition, since the temperature of the LAMP reaction (65 °C) was much lower than the highest temperature of PCR (95 °C), we found that preventing gas exchange from the sides of the chip could be eliminated in the LAMP reaction.

### 3.3. Droplet Fluorescent Detection and Counting

Before the fluorescence detection of single droplets was performed, continuous single droplet detection in the microchannel was first investigated in terms of time and space, as well as droplet size effects. For the droplet size effect, droplets with diameters of 135 μm, 100 μm, and 83 μm were produced and injected into the chip at 600 mbar. When the droplets passed through the detection point, the signal was recorded for analysis. All three droplet sizes could be distinguished and counted individually in the microchannel. In addition, the detection capability was investigated using different flow velocities of the carrying oil. Air pressures of 210, 420, 630, 840, and 1050 mbar were applied to inject droplets with a 100 μm diameter into the microchannel. The results indicated that even at 1050 mbar, the droplets could be detected and counted individually.

**Figure 5:**
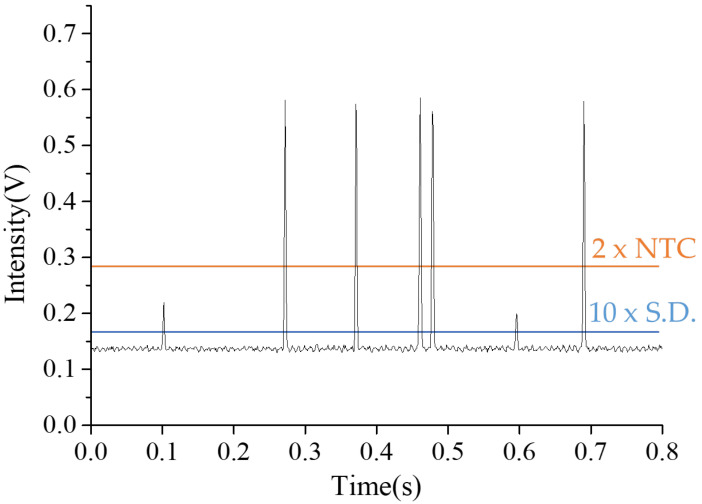
Detection of fluorescent signals from positive and negative droplets. A positive droplet contains more than one target DNA copy, allowing for the detection of a fluorescent signal after DNA replication. By contrast, a negative droplet lacks the target DNA, and any small signal detected arises from the background fluorescence of the EVA Green dye in the droplets. It is essential to set an appropriate threshold voltage to accurately differentiate between positive and negative droplets. The orange line represents the highest threshold, set at twice the average intensity of the no-template control (NTC) droplet fluorescence signals. The blue line represents the lowest threshold, determined by 10 times the standard deviation (S.D.) of the fluorinated oil signal.

Fluorescence signal detection for droplets was conducted using LabVIEW’s Threshold Peak Detector program, which sets appropriate thresholds for differentiating between positive and negative droplets. The accumulation function was used to calculate the total number of negative and positive droplets. Initially, samples containing both positive and negative droplets were prepared for testing and setting the detection conditions. Using a sample volume of 5 µL, which contained approximately 6000 droplets, a template concentration of 8.5 × 10^2^ copies/µL was selected to produce the droplets needed for fluorescence detection testing. After the droplets were formed under these conditions, the air pressure for both the oil and aqueous phases was halted, allowing the droplets to undergo the LAMP reaction in the incubation zone at 65 °C for 40 min. After completing the LAMP reaction, the oil-phase pressure was adjusted to 100 mbar to push the fluorinated oil and move the droplets to the detection zone for fluorescence signal detection. As depicted in Figure 5, the amplitudes of the fluorescence signals can be grouped into two categories: higher-amplitude signals indicate positive droplets, and lower-amplitude signals indicate negative droplets. The negative droplets likely detected fluorescence signals due to the background from the added EvaGreen dye. This background signal was used to count the total number of negative droplets. The sum of the negative and positive droplets produced the total droplet count, eliminating the need for another detection channel (such as a bright field) to count the total droplets, thereby simplifying the experimental setup for droplet detection.

The key to counting positive and negative droplets is setting thresholds. Two thresholds are necessary to distinguish between positive and negative droplets. Signals between the two thresholds are identified as negative droplets, and signals above the higher threshold are identified as positive droplets. Signals below the lower threshold are identified as noise. The lower threshold was determined based on the standard deviation of the background signal detected from fluorinated oil when no droplets were detected. Given that the fluorescence background signal caused by EvaGreen dye was not insignificant, the lower threshold was set to 10 times the standard deviation of the fluorinated oil signal. To determine high-level thresholds, we estimated that the positive droplet fluorescence signals were at least six times stronger than those of the negative droplets. Additionally, the fluorescence signal intensity for the negative droplets averaged 0.073 V (calculated as the average negative droplet peak signal minus the average noise signal from the fluorinated oil), with a standard deviation of 0.005 V. Based on these metrics, the highest threshold was set at twice the average intensity of the negative droplet fluorescence signals. By inputting these two threshold values, the quantities of positive and negative droplets could be calculated.

### 3.4. Quantitative Detection of Staphylococcus Aureus

**Figure 6:**
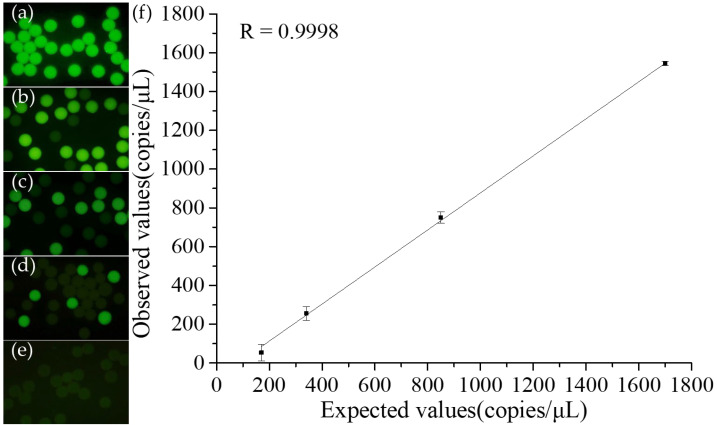
Results of digital LAMP from a tenfold serial dilution of samples performed on a microfluidic chip. The concentrations of target DNA were: (**a**) 1.7 × 10^3^ copies/µL, (**b**) 8.5 × 10^2^ copies/µL, (**c**) 3.4 × 10^2^ copies/µL, (**d**) 1.7 × 10^2^ copies/µL, and (**e**) NTC (no template control). (**f**) DNA concentration was based on the ratio of positive to total droplets compared to the expected concentration. The results closely matched the expected values.

After confirming the operational conditions and procedures for the chip droplet digital LAMP, as well as fluorescence signal detection and droplet counting, we performed quantitative detection using samples at different concentrations. We diluted *S. aureus* DNA initially at a concentration of 1.7 × 10^3^ copies/µL to 8.5×10^2^ copies/µL, 3.4 × 10^2^ copies/µL, and 1.7 × 10^2^ copies/µL. Each concentration underwent triplicate tests. Following sample partitioning, incubation, and detection on the chip, quantitative analysis was performed. We photographed the droplets after the LAMP reaction in the reservoir. Figure 6a–e shows the results for DNA concentrations of 1.7 × 10^3^ copies/µL, 8.5 × 10^2^ copies/µL, 3.4 × 10^2^ copies/µL, 1.7 × 10^2^ copies/µL, and a no-template control (NTC), respectively. We observed that the number of positive droplets decreased as the DNA concentration decreased. In addition, there was a significant fluorescent intensity difference between positive and negative droplets. Figure 6f presents the standard curve derived from the Poisson formula after counting the droplets for the four concentrations, where the x-axis represents the prepared known concentrations and the y-axis shows the calculated concentrations post-reaction. With a correlation coefficient of R = 0.9998, the platform’s quantitative results were determined to be accurate.

However, each concentration test resulted in measurements lower than the actual concentrations, and the variation was greater at 1.7 × 10^2^ copies/µL. DNA sample loss has been hypothesized to be the cause of this decrease during the partitioning process, where DNA may adhere to the centrifuge tubes or stick within the Teflon tubing and the microchannel, potentially causing undetectable levels at the lowest concentration of 1.7 × 10 copies/µL. We also used a benchtop real-time PCR machine to perform the same LAMP reaction with *S. aureus* DNA sample concentrations of 1.7 × 10^4^ copies/µL, 1.7 × 10^3^ copies/µL, and 1.7 × 10^2^ copies/µL, as shown in Supplementary Figure S3. Each concentration was tested in duplicate. The results showed that one of the 1.7 × 10^2^ copies/µL samples had a Tt value of 16.71 min, whereas the other did not amplify. By comparison, all triplicate tests of the 1.7 × 10^2^ copies/µL samples yielded detectable values using the proposed digital LAMP platform. These results confirmed that the detection limit of our microfluidic LAMP platform was more sensitive than that of the benchtop machine for LAMP reactions.

## 4. Conclusions

In this study, a single microfluidic chip was used to generate droplets that partition a sample and perform a digital LAMP reaction. The entire process was completed within 70 min, including 10 min to partition 5 µL of sample into about 6000 uniform droplets (CV = 0.82%), 40 min for the LAMP reaction in the channel, and 15 min for droplet detection and counting. The quantitative detection results were accurate, with a correlation coefficient of R = 0.9998. This study primarily addressed the issue of droplet bursting due to air contact during the LAMP reaction on the chip. Filling the channels with mineral oil and covering the chip with a glass slide helped prevent droplet–air contact. The detection limit reached as low as 1.7 × 10^2^ copies/µL. We believe that by improving issues related to DNA loss due to adhesion to the channel wall before droplets are formed, the detection limit can be further enhanced. Additionally, in the setup for detecting droplets, we explored using a fiber-optic detection framework to further miniaturize the experimental setup, facilitating the development of portable devices. This integration with the microfluidic chip allows for convenient droplet digital LAMP operations.

## Data Availability

Data are contained within the article.

## Supplementary Materials

The following supporting information can be downloaded at: https://www.mdpi.com/article/10.3390/bios14070334/s1, Figure S1: The fabrication process of the droplet-based digital LAMP microfluidic chip; Figure S2: A photograph of the assembled device indicating with each functional area; Figure S3: A benchtop real-time PCR machine was used to conduct the same LAMP reaction conditions as the chip.
