# Supplementary material for: Integrated Droplet-Based Digital Loop-Mediated Isothermal Amplification Microfluidic Chip with Droplet Generation, Incubation, and Continuous Fluorescence Detection
Source: Biosensors (Basel). 2024 Jul 8;14(7):334. doi: 10.3390/bios14070334 (PMC11275183; doi:10.3390/bios14070334)
Supplement: Supplementary file 1 [file biosensors-14-00334-s001.zip › biosensors-3012640-supplementary.pdf]

biosensors

MDPI

Supplementary Material

Integrated Droplet-based Digital Loop-mediated Isothermal Amplification Microfluidic Chip with Droplet Generation, Incubation and Continuous Fluorescence Detection

Yen-Heng Lin*, Yuan-Ting Hung, Wei Chang, Chiuan-Chian Chiou*

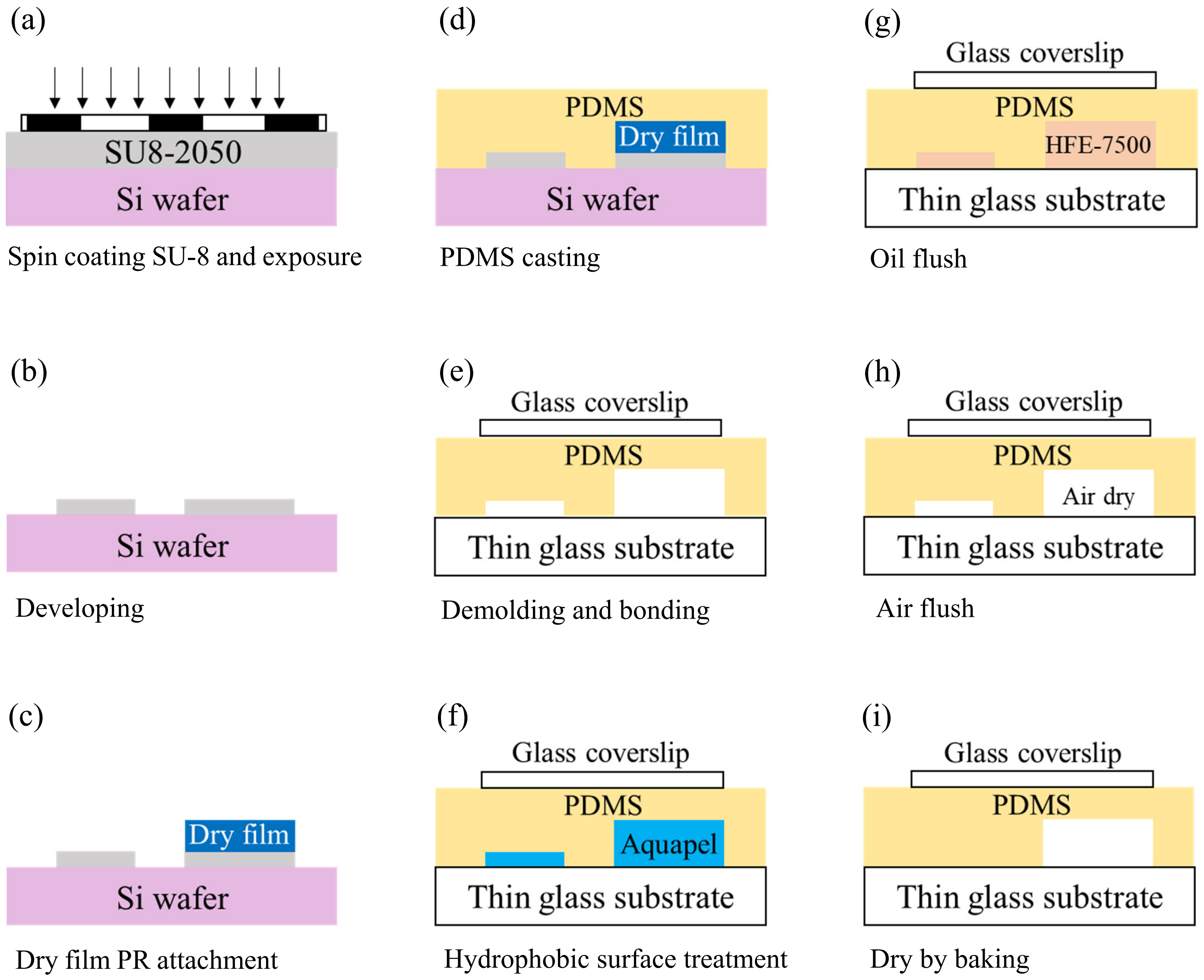

Figure S1. The fabrication process of the droplet-based digital LAMP microfluidic chip involved several steps: (a)-(c) Initially, a master mold was created using a standard photolithography process with SU-8 photoresist. To ensure an adequate volume for the waste reservoir, four layers of dry film were stacked first to achieve a height of approximately 300 μm and cut to the size of the waste reservoir and then was aligned and attached onto the SU-8 structure of the reservoir. (d)-(e) PDMS was utilized to replicate the mold structure, which was then bonded with two glass plates. One glass plate served as a substrate at the bottom of the chip, while the other was bonded on top to prevent gas penetration through the PDMS. (f)-(i) Following the formation of the microchannel, aquapel was applied to treat the microchannel walls, rendering them hydrophobic for water-in-oil droplet generation. The 200 μL of Aquapel

was injected into the microchannel using a pressure-driven pump with an applied pressure of 600 mbar. Once the microchannel was fully filled with Aquapel, another 200 μL of HFE-7500 oil was used to flush out the Aquapel at the same applied air pressure. This was followed by using compressed nitrogen gas to flush out the oil until it was completely expelled. Subsequently, the microfluidic chip was dried by baking at 80°C for over 1 hour. The chip was kept open during all surface treatment procedures. It is worth mentioning that during the hydrophobic treatment of the chip, we often found tiny precipitates stuck in the channels, especially in the cross-shaped channels with the smallest cross-sectional area, which were likely caused by the fluorinated oil or hydrophobic agent used for flushing. Therefore, we used a syringe filter (Millex-GP Filter 0.22 μm, Millipore) to filter the fluorinated oil and the hydrophobic agent Aquapel before surface modification, but still found it impossible to completely eliminate the tiny precipitates. Only after using a newly opened hydrophobic agent Aquapel for modification was the problem completely resolved, suggesting that Aquapel is very prone to reacting with air to form tiny precipitates. Therefore, in the fabrication of the chip, newly opened hydrophobic agents were always used, and the channels were flushed with filtered fluorinated oil. Detailed procedures for Aquapel treatment can be found in the following reference.

Zilionis, R.; Nainys, J.; Veres, A.; Savova, V.; Zemmour, D.; Klein, A. M.; Mazutis, L., Single-cell barcoding and sequencing using droplet microfluidics. Nature Protocols 2017, 12, (1), 44-73.

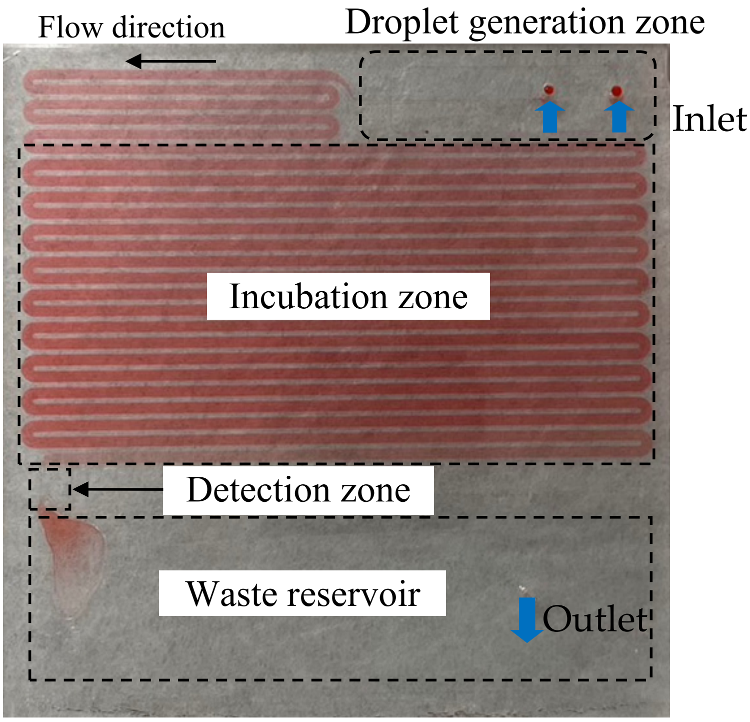

Figure S2. A photograph of the assembled device indicating with each functional area.

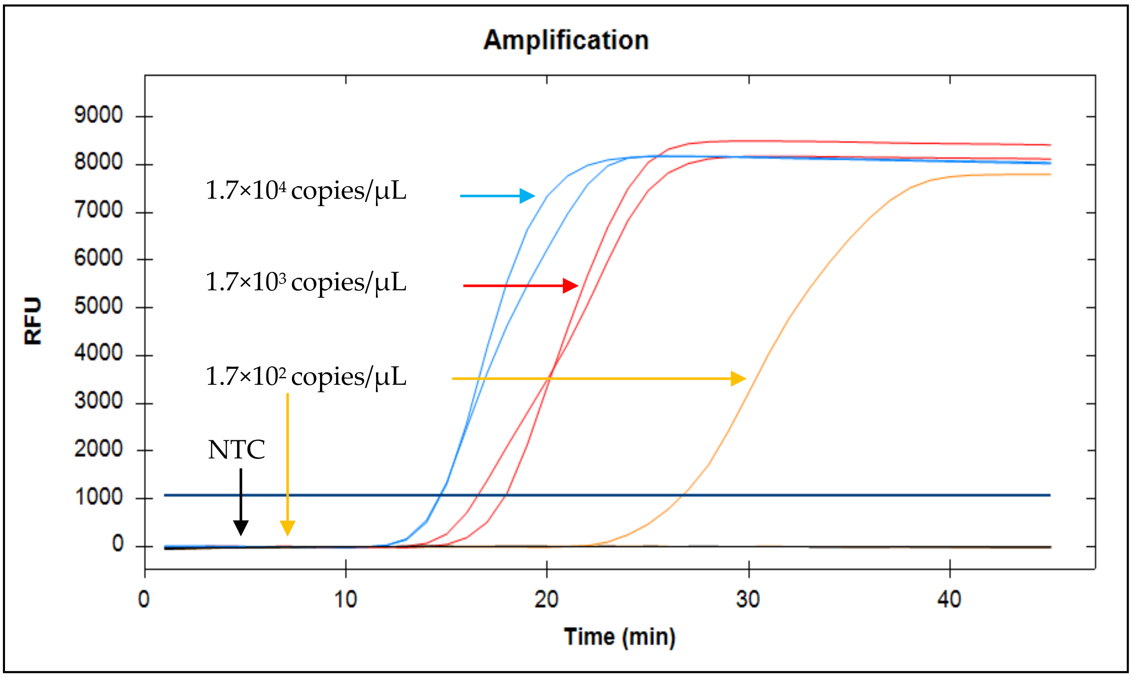

Figure S3. A benchtop real-time PCR machine was used to conduct the same LAMP reaction conditions as the chip. Three concentrations of the sample were tested, each in duplicate. The results showed that the amplification of the lowest concentration of the $1.7×10^2$ copies/μL sample could not be consistently repeated in the benchtop machine.
